# Peptide hormones and lipopeptides: from self‐assembly to therapeutic applications

**DOI:** 10.1002/psc.2954

**Published:** 2017-01-27

**Authors:** J. A. Hutchinson, S. Burholt, I. W. Hamley

**Affiliations:** ^1^ Department of ChemistryUniversity of ReadingWhiteknightsReadingRG6 6ADUK

**Keywords:** peptide hormones, lipopeptides, self‐assembly, bioactivity, peptide, therapeutics

## Abstract

This review describes the properties and activities of lipopeptides and peptide hormones and how the lipidation of peptide hormones could potentially produce therapeutic agents combating some of the most prevalent diseases and conditions. The self‐assembly of these types of molecules is outlined, and how this can impact on bioactivity. Peptide hormones specific to the uptake of food and produced in the gastrointestinal tract are discussed in detail. The advantages of lipidated peptide hormones over natural peptide hormones are summarised, in terms of stability and renal clearance, with potential application as therapeutic agents. © 2017 The Authors Journal of Peptide Science published by European Peptide Society and John Wiley & Sons Ltd.

## Peptide and Lipopeptide Self‐assembly

The self‐assembly of peptides in solution is driven by a combination of hydrogen bonding, electrostatic and other (e.g. π‐stacking) interactions that also contribute to the self‐assembly of lipopeptides 1. However, in the latter class of system, hydrophobic interactions are important. The self‐assembly of peptides and lipopeptides can be influenced by many variables including concentration, pH, ionic strength of solution or temperature 2.

Many self‐assembling molecules are amphiphilic, meaning they have both hydrophobic and hydrophilic character. They generally self‐assemble above a critical concentration, known as the critical aggregation concentration (CAC). Amphiphilic molecules such as lipids, peptides and proteins serve as building blocks for the construction of functional assemblies *in vivo*, e.g. the cytoskeleton and extracellular matrix. Lipids are one of the simplest amphiphilic structures and are composed of a hydrophilic polar head group and a hydrophobic tail. Peptides and proteins, however, are distinct in the way in which amphiphilicity is displayed because when folded, they can display regions that are either hydrophobic or hydrophilic. An example of this is an *α*‐helix, as it could contain a section of hydrophobic residues along one face and a hydrophilic section of residues on the opposite face. For *β*‐sheet structures, the peptide chain can be composed of alternating hydrophilic and hydrophobic residues, so that the side chains of the residues are displayed on opposite faces of the sheet.

**Figure 1:**
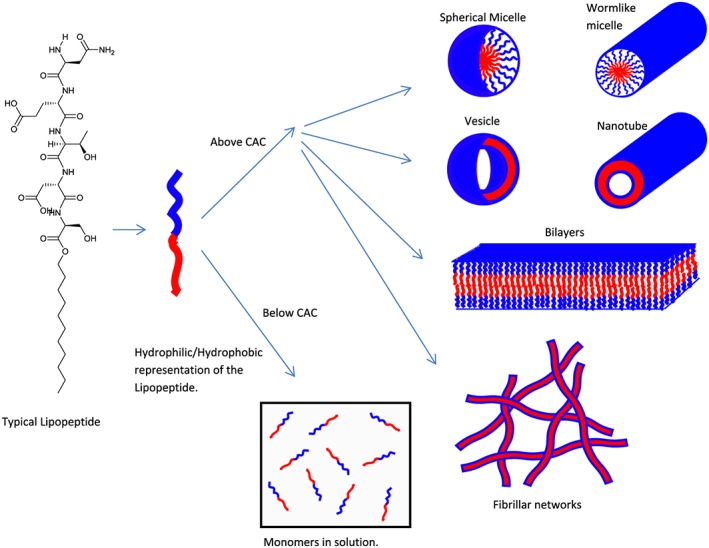
Molecular interactions and possible self‐assembled structures of typical lipopeptides.

In aqueous environments, amphiphilic molecules associate through non‐covalent interactions to form ordered assemblies of different sizes, from nanometres to microns 3. These self‐assembled structures include spherical and worm‐like micelles, vesicles, fibrils and nanotubes (Figure 1). Micelles consist of a hydrophobic inner core surrounded by a hydrophilic outer shell that is exposed to water, and they can be spheres, discs or worm‐like structures 4. Micelles form spontaneously when the concentration is above a critical micelle concentration [CMC] and temperature 5. The CMC is a subcategory of CAC, which is a more general term for the aggregation into many different structures.

Amphiphiles with an intermediate level of hydrophobicity can assemble into bilayer vesicles. Vesicles are spherical, hollow, lamellar structures with an aqueous core. The hydrophobic moieties form the inner section of the bilayer, and the hydrophilic parts are exposed to the aqueous environment 6.

Self‐assembling peptides are peptides that undergo spontaneous assembly into ordered nanostructures. This is observed depending on the hydrophilic/lipophilic balance of the molecules, as well as the interactions between the peptide units 7. In one case of self‐assembling peptides, hydrogen bonding between backbones plays an important role by forcing the peptide monomers to pack longitudinally into *β*‐sheets. The inter‐sheet interactions between the side chains of the peptides regulate lateral packing, and the stronger these interactions, the better the lateral packing 8. A further type of self‐assembling peptide forms coiled coil structures, formed from aggregated *α*‐helices 9.

Among all organic building blocks, peptides are very promising platforms because of their ease of synthesis, chemical diversity and their similar biological properties to proteins. In addition to this, peptides are very useful components in creating self‐assembled nanostructures because of their biocompatibility, biodegradability and biofunctionality 10.

The number, type and sequence of amino acids determines the self‐assembly of peptides, and depending on the amino acid sequence, the peptide can form a variety of different structures. As a result of this, peptides provide a unique platform for the design of nanomaterials with controllable structural features. Self‐assembled peptide nanostructures have demonstrated potential use for many biomedical applications such as drug delivery, tissue engineering and antimicrobial agents, to name a few, and several of these applications are discussed in detail in the following.

## Peptide Amphiphiles

**Figure 2:**
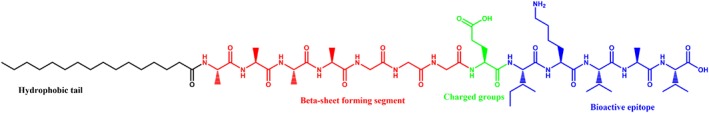
The four domains in a PA molecule required for self‐assembly into *β*‐sheet fibrils with a coating of bioactive epitopes 11.

Peptide amphiphiles (PAs) may comprise sequences of hydrophobic and hydrophilic peptides or hydrophilic peptides attached to lipid chains. The class of PA termed lipopeptide consists of one or more lipid chains attached to hydrophilic peptide sequences containing charged residues 2, 7. They are a class of molecules that combine the structural features of amphiphilic surfactants with the functions of bioactive peptides, and they are known to assemble into a variety of nanostructures 11, 12. It is proposed that PAs designed to form bioactive fibrils should be composed of four key structural features (Figure 2): A hydrophobic domain that is typically an alkyl chain attached to a peptide sequence, which favours intermolecular hydrogen bonding. Then there is a charged amino acid domain that enhances solubility in water. The final structural feature that makes up a peptide amphiphile is a bio‐derived or bio‐inspired epitope that allows interaction with cells or proteins 13.

Molecules that contain both polar and non‐polar elements often undergo self‐assembly, which allows the hydrophilic moieties to be exposed to the aqueous environment and the hydrophobic moieties to be shielded from the aqueous media. There is normally a distinct relationship between the amphiphilic character of a peptide and its function 12. For example, amphiphilic peptides fold into helices or sheets to allow the non‐polar residues to interact with the lipid chains in the interior of the cell membrane and to allow the polar residues to be exposed to the aqueous environment. This self‐assembly allows the peptide molecules to optimise their interaction with the surroundings.

Peptide amphiphiles have great potential in biomedical applications 1, 11, 13, 14, 15, 16, 17, 18 and can be utilised to act as therapeutic agents to treat diseases by delivering drugs. They can then be metabolised into lipids and amino acids, which are then easily cleared by the kidneys 19. In therapeutic applications, the hydrophobic tail assists transport across the cell membrane, and the peptide epitope can then be used to target a specific cell via a ligand‐receptor complex 20.

Lipopeptides are able to form supramolecular nanostructures such as fibrils, micelles and vesicles. Lipopeptide biosurfactants (surfactants of biological origin) are produced by a wide variety of bacteria, fungi and yeast 7, 21. They are surface active compounds that have the ability to decrease the surface and interfacial tension, allowing them to disrupt biological activity as part of the organism's host defence mechanism 21.

## Applications of Self‐assembled PAs and Lipopeptides

Lipopeptides have a wide range of applications such as use as antimicrobial agents and in immune disease therapies, cosmeceuticals and also fungicides, all of which are explained in more detail in the succeeding sections.

### Biosurfactants with Antimicrobial and Antifungal Applications

There are many types of lipopeptides; among the most popular are the classes of surfactins, iturins and fengycins, which are produced by the *Bacillus subtilis* family 21, 22, 23. *B. subtilis* strains produce a wide range of lipopeptides that are potent biosurfactants and have specific antimicrobial and antiviral activities 21. The fact that surfactins are biosurfactants means that they have diverse functional properties such as low toxicity, biodegradability and a higher tolerance towards variation of temperature and pH 21. Iturins are pore‐forming lipopeptides with antifungal activity, and this is dependent on the interaction with the cytoplasmic membrane of the target cells 21, 22, 24. Finally, fengycins are another class of biosurfactant with antifungal properties 23, 24.

### Toll‐like Receptor Agonists

Lipopeptides, such as those incorporating the CSK_4_ peptide motif and one, two or three palmitoyl (hexadecyl) chains 25, can also act as toll‐like receptor (TLR) agonists, which have important applications in the treatment of disease. TLRs are transmembrane proteins that are very important in the immune system and as a result are therapeutic targets to treat disease.

These receptor agonists respond to invading pathogens by recognising specific pathogen‐associated microbial patterns (PAMPs) or danger‐associated molecular patterns (DAMPs), which are primarily produced by microbial pathogens 26. PAMPs can contain a variety of different components including lipopolysaccharide, peptidoglycan, lipopeptide and bacterial DNA. DAMPs can be intracellular proteins or proteins from the extracellular matrix 27, 28.

Toll‐like receptors have a common structure being type‐1 transmembrane proteins. This includes having an extracellular domain formed from leucine‐rich repeats (LRRs) and a cytoplasmic tail containing an area known as the TLR domain. X‐ray crystallography reveals that the LRR domain has a horseshoe‐like shape 29, traversing the membrane. Currently, 10 TLRs have been found in humans, each with a different role and target 30.

Toll‐like receptor agonists are compounds that stimulate these receptors to modulate the interaction with one of the PAMPs/DAMPs described earlier. The development of synthetic or natural agonists is an interesting avenue of research to treat a multitude of conditions that includes, but is not limited to; advanced melanoma 31, alcoholic chronic liver disease 32, asthma 33, neuropathic pain 34 and restenosis (re‐narrowing of the blood vessels) 35.

### Skincare

**Figure 3:**
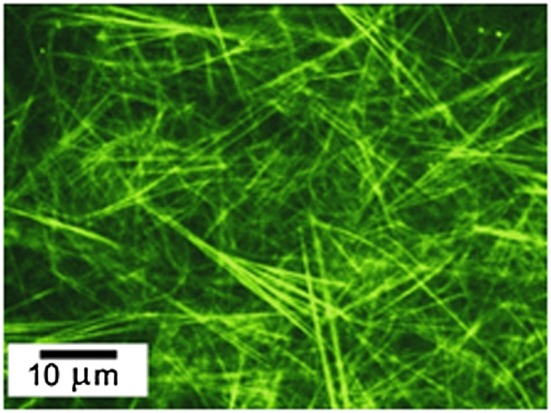
Confocal microscopy image of Matrixyl™ fibre superstructure. (Labelled with the dye rhodamine B, 0.0014 w% Matrixyl™ in water) 36.

Peptide amphiphiles are employed in skincare products with the claimed ability to help stimulate collagen production. A commercially available lipopeptide with the trade name Matrixyl™ [C_16_‐Lys‐Thr‐Thr‐Lys‐Ser (KTTKS)] has been used in anti‐wrinkle creams. This lipopeptide has been shown to self‐assemble into a *β*‐sheet tape‐like superstructure (Figure 3). Small‐angle X‐ray scattering further established a bilayer structure with a spacing of 5.3 nm 36.

The mechanism for this stimulation of collagen expression is not fully understood, but it has been reported that the KTTKS polypeptide increases the skin's extracellular matrix (ECM) production 37, 38. ECM is the outer region of a cell that supports the cells and those around it. The conjugation of the KTTKS peptide motif to a palmitoyl (C_16_) chain has been shown to enhance skin permeability, and the self‐assembled structure (Figure 3) presents a peptide‐rich surface on the nanotapes, these features contributing towards increased collagen production making it a highly sought after skincare additive. This lipopeptide's collagen‐stimulating activity occurs even at low concentration, close to its 37. Other lipopeptides, such as C_16_‐GHK or C_16_‐KT, also have been reported to have collagen‐stimulating effects, and their self‐assembly has been examined 39.

### Tissue Scaffolds

**Figure 4:**
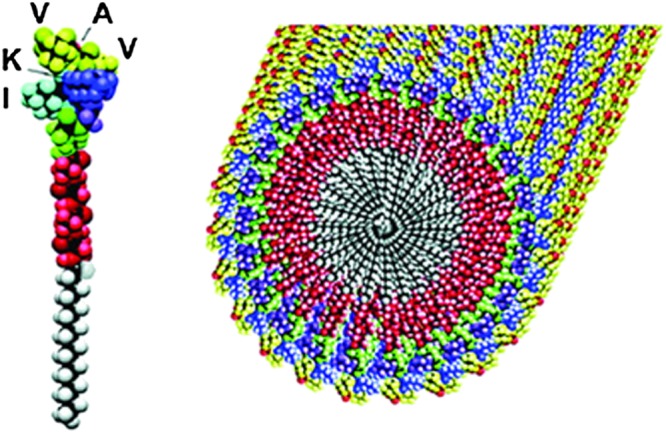
Self‐assembly of IKVAV functionalized PAs into fibrils 42.

Various groups have looked into the use of PAs and lipopeptides as scaffolds for the production of tissue or for other applications in regenerative medicine. In one study, the linear RGD amino acid sequence was conjugated with dialkyl chains. This scaffold allowed for spreading of melanoma and endothelial cells when it was carboxyl‐coupled, but spreading was not seen with amino‐coupled dialkyl chains 40. Another group managed to encapsulate cells within nanofibrils made up of PAs with the Ile‐Lys‐Val‐Ala‐Val (IKVAV) peptide group (Figure 4). These PAs were shown to be effective at treating spinal cord injuries in mice. This was due to the nanofibrils inhibiting scar formation, which helped lessen the injury 41. This is a fascinating and broad topic that has been reviewed in much detail elsewhere by the Stupp group and is therefore outside the scope of the present review 42, 43, 44, 45, 46, 47.

### Antimicrobial Materials

**Figure 5:**
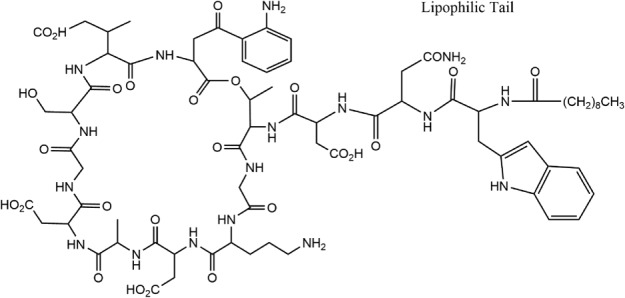
Daptomycin chemical structure, redrawn from 53.

There are a great number of studies on peptides with antimicrobial properties 48, 49, 50. A common feature for antimicrobial activity is the presence of cationic residues such as lysine or more especially arginine as these can interact with cell walls. Lipidation of peptides has been shown to improve the uptake of the peptide into the cell 51. This was investigated with a range of Gram‐negative/positive bacteria and two fungal strains. Uptake into the cell wall and then subsequent disruption of the cell membrane was found out to be the mode of action, which caused bacterial leakage and cell death 51. A peptide containing a trans‐activating transcriptional activator (TAT) sequence along with an amphiphilic Gly–Arg sequence conjugated to cholesterol was also found to be an effective antimicrobial agent 52. This peptide was shown to work by the same process of incorporation into the cell membrane followed by its disruption. The paper also showed that the PA can also be transported across the blood–brain barrier 52. One of the most well‐known lipopeptides used in treating infections is daptomycin (Figure 5) 53.

Daptomycin is able to combat systemic and life‐threatening infections and is trademarked under the name Cubicin. It comprises a cyclic peptide group joined by an amide linkage to a lipid chain. The mechanism by which daptomycin acts on bacteria has been carefully examined 54. It works by insertion of the lipid chain into the cell wall. Daptomycin molecules then aggregate, deforming the curvature of the membrane, causing holes to form and leading to the leakage of ions from the cell. This then causes a serious depolarization resulting in the inhibition of various synthesis processes including those of DNA, protein and RNA. This combines to cause cell death. The self‐assembly of daptomycin was studied outside of the cell and it was shown to aggregate into micelles 53. This aggregation process may be correlated to its excellent antimicrobial activity. A recent review on the self‐assembly of lipopeptides including therapeutic lipopeptides is available 7.

### Drug Delivery

Lipopeptides are interesting vehicles for drug delivery with several modes of activity. In one, the self‐assembled structure of the PA is exploited, i.e. the hydrophobic area inside a micelle or bilayer structure is used to house a hydrophobic drug molecule. It is important to distinguish between PAs with inherent therapeutic potential and those that are merely vehicles or devices. In the former case, many types of bioactive peptides have been lipidated to create active pharmaceutical ingredients (APIs) (e.g. Table 1). In the latter case, the self‐assembled structure allows for the transport of the drug in highly aqueous environments to the target cells. An example of this is a TAT_48–60_ fragment conjugated with one, two or four attached octanoic acid groups 55. These TAT PAs form β‐sheet structures. It was shown that the PA with four octanoic acid groups could encapsulate and retain the hydrophobic drug paclitaxel. This was deemed to be due to the high hydrophobicity of the octanoic acid groups. This encapsulation was highly efficient (6.8±0.4%), compared with previous nanoscale delivery vehicles for which encapsulation rarely exceeds 5%, making it a favoured drug delivery system 55. Another strategy to create PAs for drug delivery is the lipidation of receptor‐specific peptide head groups. The peptide head groups bind selectively to the receptor, while the lipid group allows the PA to cross cell membranes and also increases bioactivity through reducing metabolic degradation 56, 57. A good example of this is an amphiliphic lipopeptide comprising a palmitoyl (C_16_) ester linked to Ala‐Gly‐Phe‐Leu‐Arg peptide motif, incorporating the anticancer drug Dalargin (Ala–Gly–Phe–Leu–Arg), which formed into nanofibres 58.These fibres have high circulation times and also more importantly can cross the BBB. This allows the normally hydrophobic cancer drug to potentially be used to treat cancers in the brain, overcoming its low circulation and high hydrophobicity 58.

**Table 1:** Peptide therapeutics on the market

Trade name	Peptide	Company	Molecular properties	Related reference
Copaxone	Glatiramer	Teva	Four amino acids (l‐glutamic acid, l‐alanine, l‐lysine and l‐tyrosine) in a defined molar ratio	105, 106
Lupron	Leuprolide	Abbott	Synthetic nonapeptide analogue of naturally occurring gonadotropin‐releasing hormone (GnRH or LH‐RH)	107, 108
Vicoza	Liraglutide	Novo	97% homologous to native human GLP‐1 (7–37) by substituting arginine for lysine at position 34 and addition of a fatty acid chain	109
Zoladex	Goserelin	AZ	Natural LHRH/GnRH decapeptide with two substitutions to inhibit rapid degradation.	110, 111
Sandostatin	Octreotide	Novartis	Longer acting synthetic octapeptide analogue of naturally occurring somatostatin	112, 113
Forteo	Teriparatide	Lilly/Amylin	Recombinant form of parathyroid hormone consisting of the first (N‐terminus) 34 amino acids, which is the bioactive portion of the hormone	114, 115
Byetta	Exenatide	Lilly/Amylin	Synthetic version of exendin‐4, a hormone found in the saliva of the Gila monster	116, 117
Cubicin	Daptomycin	Cubist	Cyclic lipopeptide, consisting of 13 amino acids, 10 of which are arranged in a cyclic fashion and three on an exocyclic tail	118, 119
Integrilin	Eptifibatide	Merck	Cyclic heptapeptide composed with S–S bridge, two unnatural building blocks and amide	120, 121
Angiomax/angiox	Bivalirudin	Medicines	20‐amino acid polypeptide	122, 123
Fortical	Calcitonin	Upsher‐Smith	32‐amino acid polypeptide	124, 125, 126
Somatuline	Lanreotide	Ipsen	Cyclic peptide that is a long‐acting analogue of somatostatin.	127, 128

### Biomaterial Templating

**Figure 6:**
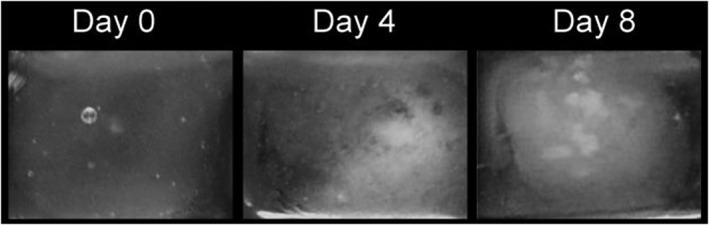
Time‐sequence photographs of a mineralising 3D PA matrix 61.

The various structures formed by PAs and lipopeptides have been exploited to template distinct inorganic materials (salts, metal oxides and metals), sometimes allowing for unique structures to be formed. PA nanostructures have the potential, for example, to be used to template the bone mineral hydroxyapatite, which is the body's primary storage depot for calcium and phosphorus in bones 59, 60. A further avenue of interest is the incorporation of other metals, such as titanium, with mineralizing PAs to form hybrid bone implants. In one example, a titanium alloy foam was mixed with different PAs solutions and then further allowed to mineralise calcium phosphate. This was shown (Figure 6) to form new, immature bone adjacent and inside the hybrid, after 4 weeks 61. This field of research is highly active and may, in the future, allow for treatment of bone diseases such as hydroxyapatite deposition disease or dystrophic calcification, among others.

## Peptide Hormones

Peptide hormones are hormones made up of amino acid chains that primarily have an effect on the endocrine system. Based on the building units, hormones can be classified as either amino acid‐based or steroid‐based systems. The presence of amino acids in peptide hormones allows them to act on the surface of target cells via secondary messengers. This differs from steroid hormones that are lipid soluble, and so can move through the plasma membranes of target cells and act within the nuclei 62.

The endocrine system is composed of many different glands, and it can be divided into two categories: classical and non‐classical. In the endocrine system, hormones are secreted into the circulatory system where they are distributed throughout the body, regulating bodily functions. The classical endocrine glands include the pituitary gland, pancreas, thyroid gland, adrenal cortex and medulla. The primary function of these glands is to manufacture specific hormones. Non‐classical endocrine glands include the heart, hypothalamus, kidneys, liver and the gastrointestinal tract. Many of the classical hormones are controlled by the hypothalamus and pituitary, which can also be classified as being an extension of the nervous system 63.

## Gut–Brain Interactions

**Figure 7:**
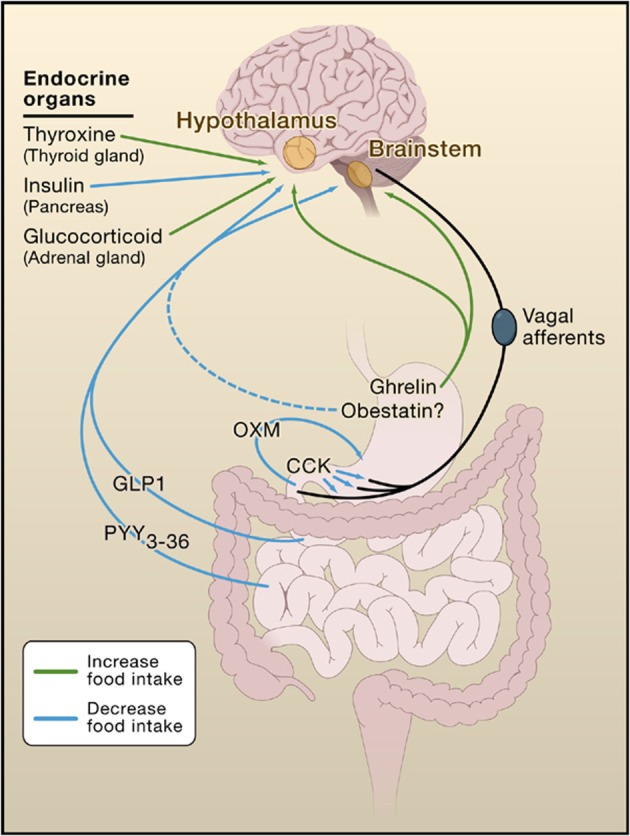
Interactions of gut and endocrine hormones with the brain and how they affect food intake 66.

Gut–brain interactions are increasingly recognised as playing an important role in determining overall food intake 63. Many peptides are synthesised and released from the gastrointestinal tract, and it has been shown that they physiologically influence eating behaviour via gut–brain signalling 64. Ghrelin is an appetite‐stimulating peptide produced in the stomach, which acts as a meal initiator. This differs from peptide YY, pancreatic polypeptide (PP), glucagon‐like peptide‐1 (GLP‐1), oxyntomodulin (OXM) and cholecystokinin (CCK), which are all derived from the intestine and pancreas and have been shown to produce satiety signals. From this, it has been suggested that gut hormones can be manipulated to regulate energy balance, and as a result, gut hormone‐based therapies could be a possible treatment for obesity (Figure 7) 65, 66.

## Peptide Hormones Involved with the Gastrointestinal Tract and Feeding

### Leptin

Leptin is a hormone made by adipose cells that affects many biological mechanisms including reproduction, the immune and inflammatory response, haematopoiesis, angiogenesis, bone formation and wound healing. More interestingly, however, leptin helps to regulate energy balance by inhibiting hunger. This occurs via a feedback mechanism in which signals are sent to key regulatory centres in the brain to inhibit food intake 67.

After leptin is released by the adipose tissue into the bloodstream, it crosses the BBB and binds to the hypothalamic leptin receptors. This affects the activity of many hypothalamic neurones and the expression of various orexigenic (appetite stimulating) and anorexigenic neuropeptides. Orexigenic peptides include neuropeptide Y (NPY), and anorexigenic peptides include pro‐opiomelanocortin. It has been suggested that the interaction with both types of these neuropeptides underpins the mechanism of action of leptin in the hypothalamus to inhibit hunger 67.

### Ghrelin

Ghrelin is a 28‐amino acid peptide with an octanoylated serine residue at position 3 68 and is produced and secreted by cells within the oxyntic glands of the stomach 65. Peripheral administration of ghrelin has been shown to stimulate food intake and decrease fat utilisation. This means it is involved in energy homeostasis, and it is the serine residue that appears to give ghrelin these effects 68. What makes ghrelin unique is its function to increase food intake rather than decrease it, and as a result, it is a very important component of weight control. Evidence of this was shown when a study was carried out on mice that were lacking in ghrelin. The results showed that they were resistant to diet‐induced obesity when fed a high‐fat diet due to them eating less, and therefore utilising more stored fat as an energy source 66.

### Cholecystokinin

Cholecystokinin is an endogenous gut hormone mainly found in the duodenum and jejunum, which exists in several molecular forms with differing numbers of amino acids. Examples include CCK‐8 and CCK‐54 (the number indicates the number of amino acid residues). CCK is known to act as a postprandial satiety signal, and it acts via two receptors: CCK_1_ and CCK_2_. The CCK_1_ receptor is more important in appetite control 68. The receptors are located on the peripheral vagal afferent terminals, which transmit signals to the part of the brain stem that is associated with appetite, such as the nucleus of the solitary tract 66.

### Oxyntomodulin

Oxyntomodulin is a 37‐amino acid peptide expressed in the central nervous system and the L cells of the intestine and pancreas 69. OXM seems to mediate its effects via the GLP‐1 receptor as shown in experiments carried out on rat parietal cells 70. This has been proven since its anorectic actions are blocked when the GLP‐1 antagonist was administered 71. Intravenous administration of OXM in humans inhibits gastric emptying and gastric acid secretion, which leads to a feeling of satiety 72. This feeling of satiety can cause a reduction in both food intake and overall body weight, and this is brought about by the suppression of ghrelin.

### Glucagon‐like Peptide‐1

Glucagon‐like peptide‐1 is a 30‐amino acid gut‐derived incretin peptide hormone 73 meaning that it stimulates insulin secretion in response to eating, and as a result, it suppresses glucagon secretion. In addition to this, GLP‐1 inhibits gastric emptying and also reduces appetite and food intake 74. GLP‐1 is produced in the intestinal epithelial endocrine L cells in the distal small bowel and colon by differential processing of proglucagon 73, 74. Proglucagon is the gene that is expressed in the L cells and is regulated in the gut and brain 74. Within minutes of food intake, the plasma levels of GLP‐1 rise rapidly. GLP‐1 exists in two circulating molecular forms: GLP‐1(7–37) and GLP‐1(7–36) amide, and it is GLP‐1(7–36) amide that represents the majority of circulating active GLP‐1 in human plasma. Both forms of GLP‐1 are rapidly metabolised and inactivated by the enzyme dipeptidyl peptidase‐4 (DPP‐4) to GLP‐1(9–37) or GLP‐1(9–36) amide following the release from gut L cells 75. This widely expressed enzyme cleaves both forms of GLP‐1 at the position 2 alanine of the N‐terminal to make them inactive. The expression of DPP‐4 in the gut and vascular endothelium explains the short half‐life of GLP‐1 of just several minutes, because the majority of immunoreactive GLP‐1 entering the portal venous circulation has already been inactivated by N‐terminal cleavage 76.

### Pancreatic Polypeptide

Pancreatic polypeptide is a 36‐amino acid peptide that belongs to a family that includes NPY and peptide YY (PYY), and all of these peptides are members of the PP‐fold peptide family. The PP‐fold family binds to receptors Y_1_–Y_6_, but PP in particular has the highest affinity for the Y_4_ and Y_5_ receptors 68. PP is similar to GLP‐1 in that it is released into the circulation after the ingestion of food. However, it differs in that it is produced in the endocrine F cells, which are located in the periphery of the pancreatic islets 77, 78. PP is responsible for a number of regulatory actions, such as the inhibition of pancreatic exocrine secretion, and the modulation of gastric acid secretion, and gastric emptying 79, 80. The amount of PP released is affected by the digestive state, i.e. release is very low in the fasted state but is significantly increased throughout all phases of digestion. In addition to this, PP is affected by a decrease in blood glucose levels and insulin‐induced hypoglycaemia, both being stimuli for PP secretion in the brain. As a result of this, it is thought that PP could potentially play a significant role in the regulation of feeding behaviour to control energy homeostasis 77.

### Peptide YY

Peptide YY is a gut hormone that belongs to the PP family, along with PP and NPY, all of which are given the term PP‐fold family. The PP‐fold motif is found throughout this family and relates to the 3D structure. The PP‐fold is formed through the incorporation of certain residues, which are predominately Pro2, Pro5, Pro8, Gly9, Tyr20 and Tyr27. The PP‐fold has been found to protect the peptide against enzymatic attack as well as producing a hydrophobic pocket that is inherently overall energy‐reducing. In addition to containing the PP‐fold motif, PYY and its derivative PYY_3–36_ also have a high C‐terminal *α*‐helix content, which has also been suggested to be extremely important for the structural integrity of PYY. The sequence of the 36‐amino acid peptide, PYY, is Tyr–Pro–Ala–Lys–Pro–Glu–Ala–Pro–Gly–Glu–Asp–Ala–Ser–Pro–Glu–Glu–Leu–Ser–Arg–Tyr–Tyr–Ala–Ser–Leu–Arg–His–Tyr–Leu–Asn–Leu–Val–Thr–Arg–Gln–Arg–Tyr–NH_2_ 81.

**Figure 8:**
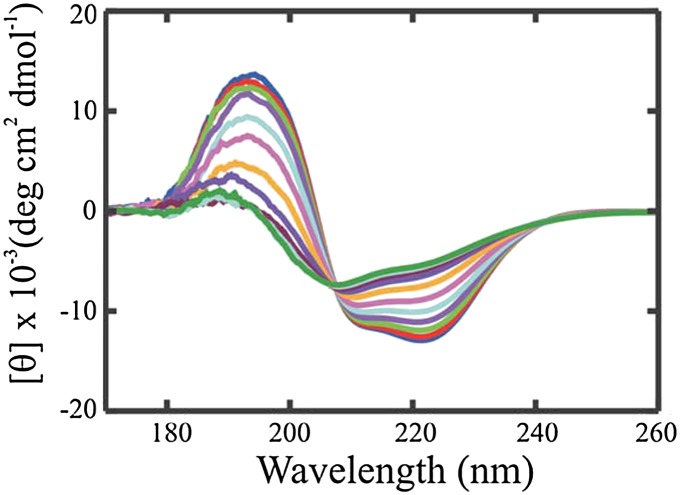
Temperature dependant CD spectra of human PYY in 20 mM acetate buffer at pH 4.6 82.

Temperature‐dependent circular dichroism (CD) studies have been carried out on human PYY to characterise its secondary structure. The results indicated an *α*‐helical structure due to a maximum near 190 nm and two minima near 208 and 222 nm. A minimum near 200 nm was also observed, which corresponds to a random‐coil structure (Figure 8) 82.

The PP‐fold PP family all consists of a signal peptide, followed by a 36‐amino acid active peptide and a carboxyl‐terminal 83, and they mediate their effects through the NPY receptors Y_1_, Y_2_, Y_4_ and Y_5_ 84. The Y‐receptors belong to the G protein‐coupled receptor family, and they mediate a wide variety of physiological effects such as regulation of blood pressure, anxiety, memory retention, hormone release and food intake.

**Figure 9:**
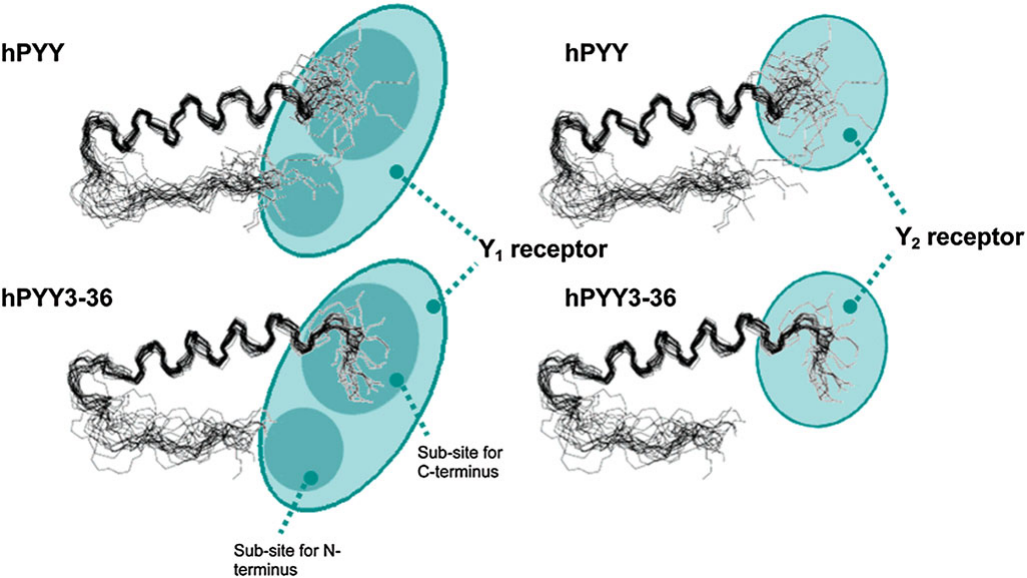
Binding sites for Y1 and Y2 with hPYY + hPYY_(3–36)_ 86.

Peptide YY is released by the L cells of the gastrointestinal tract following food intake, and there are two main endogenous forms: PYY_1–36_ and PYY_3–36_. PYY_1–36_ is rapidly processed by the enzyme DPP‐4 to the 34‐amino acid peptide PYY_3–36_ 85. DPP‐4 hydrolyses PYY and removes the first two amino acids, tyrosine and proline, at the N‐terminal, which changes the receptor selectivity. As a result of this, PYY_3–36_ has a high selectivity for the Y_2_‐receptor, compared with PYY_1–36_, which has selectivity for the Y_1_, Y_2_ and Y_5_ receptors. It is thought that the Y_1_ receptor requires both the C‐terminus and N‐terminus for recognition, binding and then subsequent activation. The Y_2_ receptor is thought to have a smaller receptor site and also only requires the C‐terminus for recognition (Figure 9).

This could explain the reduced affinity for PYY_3–36_ on any Y receptor other than Y_2_ 86. Other studies replacing the amide bonds with ester bonds also confirm that the end section is important in binding and activation 87. The Y_2_ receptors are located in the hippocampus, sympathetic and parasympathetic nerve fibres, intestines and certain blood vessels and have been implicated in regulating food intake and gastric emptying 88. As a result of this, the Y_2_ receptor is considered a target for the treatment of obesity and type 2 diabetes.

### Neuropeptide Y

Neuropeptide Y is also a 36‐amino acid peptide that has a very similar sequence homology to PYY and PP. NPY, however, differs from the other two peptides in the fact that it acts as a neurotransmitter rather than a hormone 89. NPY is one of the most abundant peptides found in the brain 90, and it is synthesised and released by neurons, which in the peripheral nervous system are mostly sympathetic neurons 89. NPY is associated with various biological responses, which include increased food intake, enhanced cognitive function associated with learning and memory and also reduction in anxiety 91. In addition to this, in peripheral blood vessels, NPY has been shown to induce vasoconstriction 92. Studies of NPY and its receptors suggest that it could be directly related to various pathological disorders such as obesity, depression and epilepsy 93.

## Peptide Therapeutics

There are many peptide products on the market at present, and this are likely to increase because of their specificity, potency and low toxicity 94, 95. Table 1 shows some of the leading peptide therapeutics. Not all of these peptide therapeutics self‐assemble or are formulated as solutions, but the table shows examples highlighting the importance of the use of peptides in drugs. The highest selling marketed diabetic drug Liraglutide incorporates a lipid chain to extent plasma circulation and ensures prolonged bioavailability 96, 97. Liraglutide is a GLP‐1 agonist drug that self‐assembles into an *α*‐helical structure, and it requires once a day administration 98. Lipid conjugation of a palmitoyl chain to a lysine residue at position 26 of liraglutide results in an extended half‐life (around 13–14 h) in the blood. This is due to the palmitoyl chain allowing non‐covalent binding to albumin, which delays proteolytic attack by DPP‐4 and also rapid renal clearance. Furthermore, the addition of the lipid chain could further prolong half‐life by sterically hindering the DPP‐4 enzyme from degradation 99.

Another peptide known to self‐assemble is the octapeptide Lanreotide. This compound is a synthetic analogue of the peptide hormone somatostatin, and it is used to treat acromegaly 100 (a condition where the body produced too much growth hormone). In water, lanreotide self‐assembles into monodisperse liquid crystalline nanotubes. The nanotubes are made up of dimers that self‐assemble into a 2D crystal, which is held together by lateral chain interactions within antiparallel *ß*‐sheets 100, 101.

Daptomycin is a lipopeptide therapeutic agent that is used as an antibiotic to treat infections caused by Gram‐positive bacteria 102. This drug is particularly effective against infections that are antimicrobial resistant such as MRSA. The antimicrobial activity of daptomycin is calcium‐dependent. In the presence of a 1 : 1 ratio of calcium ions to daptomycin, self‐assembly into micelles has been observed. The mechanism of action involves the disruption of the negatively charged bacterial cell membrane, in the presence of cations (for antibiotic activity, this is calcium ions) 53.

A further example of how self‐assembly and bioactivity of peptide hormones are related involves self‐assembling amyloid structures formed by peptide hormones and neuropeptides, which are crucial messenger molecules responsible for the function of different cells and organs. Peptide hormones and neuropeptides form aggregates that pack into dense‐core vesicles (DCVs), which are used to temporararily store peptide messengers in secretory cells 103. When DCVs are triggered, they release the stored contents into the blood or extracellular space 104, which results in amyloid disassembly, in order for action 103. Therefore, for these types of peptides, reversibility of peptide aggregation is essential for their function.

As mentioned earlier, Table 1 shows some examples of peptide agents that are currently on the market.

## Therapeutic Applications of PYY Peptides

A significant amount of research into the effects that PYY and PYY_(3–36)_ have on the body has been undertaken, predominately in the food/biological science fields 129, 130, 131. The application of PYY_(3–36)_ as an anti‐obesity drug is an extremely interesting topic of research; however, it is limited because of its short half‐life and lack of selectivity for the Y_2_‐receptor 85. As a result, there is an unmet need for a treatment, and because peptide hormones already exist, *in vivo* peptide‐based drugs are very favourable compared with synthetic molecules. This is due to them being less toxic, more selective and also more predictable in their *in vivo* behaviour 132. In contrast, however, the use of peptides and proteins as therapeutic agents has its drawbacks because of their rapid degradation, excretion, and poor water solubility. In addition to this, they may cause allergic reactions due to immune responses. However, this applies more to proteins than peptides as peptides are generally less immunogenic. One of the main reasons for the short half‐life of peptides is due to their rapid renal excretion, and this can be overcome by increasing the molecular weight (upper limited >2000 gmol^−1^). A strategy to do this is to PEGylate the peptides. Covalently attaching PEG chains to the peptide improves enzymatic stability because of steric hindrance of proteolytic enzymes. This suppressed the immune response 132, 133, 134. PEGylation has been shown to induce vacuolisation (formation of vacuoles within/adjacent to cells), but current research has focused on limiting this effect by using different carriers or shortening chain lengths 135, 136, 137. In addition to extending half‐life, PEGylation exhibits many properties that are favourable in pharmaceutical applications such as high water solubility, low toxicity and immunogenicity and ready clearance from the body. An example to show the effectiveness of PEGylation on half‐life is the study by Lee *et al.*, where site‐specific mono‐PEGylation of GLP‐1 led to a 16‐fold increase in plasma half‐life time in rats 138.

As previously mentioned, the ability of PYY and PYY_3–36_ to reduce food intake makes this peptide promising for use in anti‐obesity or chronic eating disorder medications. A few studies have shown that even though PYY works as an appetite suppressant, it has some drawbacks that need to be overcome. A few studies that used high doses of PYY have reported taste aversion in animals and nausea in humans 139, 140. This was compounded by rapid dose administration. These side effects were combated by low doses at a steady and controlled infusion mainly through intravenous injections 141. The two main problems with the administration of PYY seem to be both its concentration and how it is given, with the optimum dose being obtained by intravenous administration 142. This is a detriment for the patient as they have to inject themselves, potentially daily, during the programme, and this can cause problems. Oral or nasal delivery gets over these issues, but with potential efficacy issues due to uptake 143, 144.

A paper in 2002 commented on the potential for acute peripheral administration of PYY_(3–36)_ to inhibit food intake. PYY_(3–36)_ was injected into fasted (for 24 h) rats' hypothalamuses, and this led to a significant decrease in food intake, even at doses as low as 100 μg kg^−1^ 145. This effect was initially controversial with very few other researchers being able to repeat the result 146. A theory behind the controversy was proposed, this being that the mice used in the repeat tests were unaccustomed to the laboratory environment, which increased their stress levels. Stress inherently reduces baseline food intake so it makes any investigations in anorectic agents difficult to assess 146. Further research into the use of PYY_(3–36)_ in human studies has found that obese subjects show normal levels of sensitivity to the appetite‐reducing effects 147.

## Summary and Conclusions

Peptides have many advantages as therapeutic agents. This is due to them being multifunctional and having a diverse range of applications; from antimicrobial agents to triggering a cellular release of hormones. Table 1 provides a list of examples of peptide drugs currently on the market.

Lipidation is a useful tool to reduce the degradation of a drug, or to extend half‐life, with some of these effects probably being the result of self‐assembly, although much more work is needed to investigate the possibly of many lipidated (and also non‐lipidated) peptide drugs. Lipidation also enhances interactions of peptides with cell membranes.

As discussed earlier with appropriate examples, lipopeptides and PAs have a lot of interesting applications including use in antimicrobial and antifungal treatments, as skin care product ingredients, in drug delivery systems including those for hormone diseases as highlighted herein and in material templating in tissue engineering.

Peptide hormone therapeutics are of great interest as components of ‘pre‐built’ drugs that the human body has already created and responds to. This significantly reduces the work needed to find a synthetic drug that responds to the active site. One drawback is that peptide hormones *in vivo* are stable for a specific time frame, before being removed. This may be a problem if the peptide hormone is of interest as a drug candidate, due to rapid clearance from the body. This is where lipidation may be beneficial as it can lead to a reduction in the drug clearance rate and proteolytic degradation. This offers great potential in the development of future therapeutics.

The peptide hormone PYY_3–36_ is released by the L cells of the gastrointestinal tract following food intake and has a high selectivity for the Y_2_ receptor. The Y_2_ receptor is associated with the regulation of food intake and gastric emptying. As a result of this, the use of PYY_3–36_ as a peptide hormone therapeutic to treat lifestyle conditions such as obesity and type 2 diabetes is a very promising area of research. As previously mentioned though, peptide hormones have short half‐lives and are rapidly cleared from the body, which means that their activity as therapeutic agents will be short lived, and lipidation and PEGylation are strategies to overcome this.

In summary, this review has covered applications of peptide‐based molecules, especially peptide amphiphiles and lipopeptides, with a particular focus on peptide hormones and their uses as drugs. We have highlighted the limited number of studies so far in which it has been suggested that self‐assembly may influence bioactivity. Much more research is required into this fascinating subject as novel peptide‐based molecules emerge as future therapeutic agents.
